# Biology and External Morphology of Immature Stages of the Butterfly, *Diaethria candrena candrena*

**DOI:** 10.1673/031.012.0901

**Published:** 2012-01-25

**Authors:** Fernando M.S. Dias, Eduardo Carneiro, Mirna M. Casagrande, Olaf H.H. Mielke

**Affiliations:** Laboratório de Estudos de Lepidoptera Neotropical, Departamento de Zoologia, Universidade Federal do Paraná, P.O. Box 19.020, ZIP Code 81.531 –980, Curitiba, Paraná, Brazil

**Keywords:** Callicorini, chaetotaxy, life—cycle, Sapindaceae, *Serjania*

## Abstract

The biology and the external morphology of immature stages of *Diaethria candrena candrena* (Godart) (Lepidoptera: Nymphalidae: Biblidinae) are described. Immature *D*. *c*. *candrena* found on *Allophylus* spp. (Sapindaceae) were collected in Curitiba, Paraná, Brazil and reared in the laboratory. Morphological descriptions and illustrations are given, based on observations using electronic, stereoscopic, and optic microscopes, the latter two attached to camera lucida. Results are compared and discussed with immature stages of other species of Biblidinae described to date.

## Resumo

Descreve-se a biologia e a morfologia externa dos estágios imaturos de *Diaethria candrena candrena* (Godart) (Lepidoptera: Nymphalidae: Biblidinae) coletados em espécies *de Allophylus* (Sapindaceae) em Curitiba, Paraná, Brasil. Características morfológicas são descritas e ilustradas, como resultado de observações em microscõpios eletrônico, estereoscópico e ótico, os dois últimos acoplados à câmara clara. Resultados são comparados e discutidos com estágios imaturos de outras espécies de Biblidinae já descritos.

## Introduction

**Figures 1–4.**
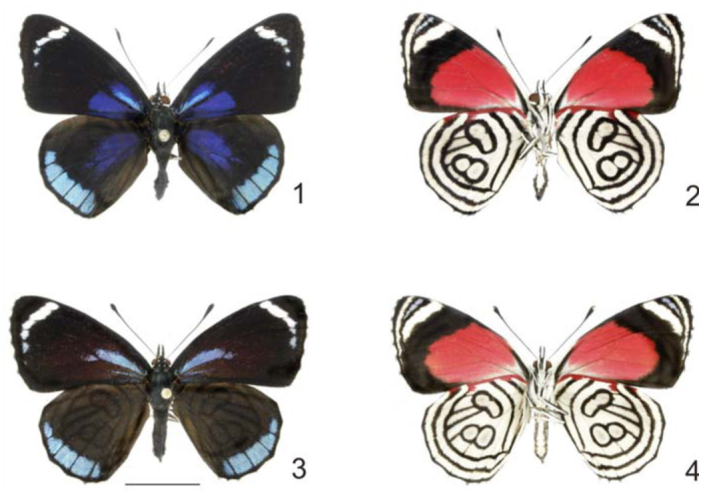
Adults of *Diaethria candrena candrena* (Godart) 1. Male, dorsal. 2. Male, ventral. 3. Female, dorsal. 4. Female, ventral. Scale bar = 1 cm. High quality figures are available online.

The Neotropical genus *Diaethria* includes twelve species, with an additional 43 subspecies (Lamas 2004); popularly known as ‘eighty’ or ‘eighty—eight’ butterflies for their suggestive hind wing underside patterns that consist of black dots or white areas surrounded by concentric black and white lines. Most *Diaethria* species occur in montane or submontane habitats of Central America, the Andes, and the Amazon Basin, with only three species occurring further south (D'Abrera 1987). *Diaethria candrena* (Godart 1824) (Lepidoptera: Nymphalidae: Biblidinae) occurs throughout South America, with two recognized subspecies (Lamas 2004): *D*. *candrena longfieldae* Talbot, occurring in the Amazon Basin south of the Amazon River; and *D*. *candrena candrena* (Godart 1824) (Figures 1–4), occurring in south and southwestern Brazil, eastern Paraguay, northern Argentina, and Uruguay (Brown 1992; Canals 2003; Betancur-Viglione 2009; Nuñes-Bustos 2009), and therefore is the southernmost taxon of the genus (D'Abrera 1987).

*Diaethria c*. *candrena* is reported to be abundant in suitable habitats like humid forests or along forest edges and streams (Brown 1992; Canals 2003). Although similar to other *Diaethria* species, *D*. *c*. *candrena* can be easily distinguished by its mostly black forewing, with basal deep blue flush and a narrow whitish band near the apex; hind wing mostly black with basal deep blue flush and broad blue band along the outer margin. Sexual dimorphism is noticeable: females (Figures 3–4) are slightly duller, with the basal blue flush reduced on the forewing and absent on the hind wing.

Even though *Diaethria* is the most diverse genus of its tribe, Callicorini, immatures of only three species were described to date: *D*. *clymena meridionalis* (Bates 1872) (Müller 1886), *D*. *clymena janeira* (C. Felder, 1862) (D'Almeida 1922; Barbosa et al. 2010), *D*. *astala* (Guérin-Méneville, (1844))(Muyshondt 1975), and *D*. *pandama* (Doubleday, (1848)) (Muyshondt et al. 1976).

Most host plant records for *Diaethria* immatures (including *D*. *candrena*) belong to species of genera *Serjania*, *Paullinia*, and *Allophylus* (Sapindaceae) (Pastrana 2004, Teshirogi 2007, Beccaloni et al. 2008), although there are reliable records for species of genera *Celtis* and *Trema* (Ulmaceae) (Brown 1992; Pastrana 2004; Beccaloni et al. 2008; Barbosa et al. 2010). Eggs are described as finely sculptured; first and second instars build frass chains, and the head capsule bears long and branched scoli from the third instar onwards (Muyshondt 1975; Muyshondt et al. 1976; Teshirogi 2007; Barbosa et al. 2010). Compared to other genera of Callicorini, fifth instar larvae of *Diaethria* show marked reduction of the size of body scoli (Teshirogi 2007; Barbosa et al. 2010). Pupae are typically green with two small lateral protuberances on the head region (Barbosa et al. 2010).

As pointed out by D'Abrera (1987), knowing the immature stages of ‘as many species as possible’ could help to settle species identity problems caused by the great deal of infraspecific and infrasubspecific variation found on *Diaethria*. Barbosa et al. (2010) reinforces that additional detailed descriptions of species already briefly described can be helpful in the identification of “stable versus variable traits”. Additionally, since immature stages are an important source of information for butterfly systematics (Freitas and Brown 2004), description of any butterfly species is especially relevant considering the small number of Biblidinae species with adequate immature descriptions (Freitas et al. 1997; Barbosa et al. 2010). Therefore, this paper comprehensively describes for the first time the biology and the external morphology of *D*. *candrena candrena*.

## Materials and Methods

Specimens studied were collected in several occasions between April 2008 and May 2009 at Parque Municipal Barigui, Curitiba, Paraná, Brazil (25^°^25′36″ S, 49^°^18′ 32″ W; ∼950 m). Collected specimens were brought to the Departamento de Zoologia, Universidade Federal do Paraná and reared in an ambient non—controlled conditions in the Laboratório de Estudos de Lepidoptera Neotropical. Specimens were reared individually in plastic containers and old leaves of the host plant were changed for fresher ones as necessary. The plastic containers were examined daily to observe instar changes and behavior, and also to control the moisture inside. Behavioral observations were carried out in the field as well as in the laboratory. As laboratory conditions did not necessarily match those of the areas where larvae were collected, the durations of life—stages reported here may not correspond exactly with natural life—cycle durations.

Eggs and head capsules were dehydrated and preserved; larvae and pupae were fixed in Kahle-Dietrich solution (Tripplehorn and Johnson 2005) and preserved in 80% alcohol. Eggs were analyzed using an scanning electron microscope (Jeol® JSM — 6360LV, www.jeol.com); chaetotaxy of head capsule was observed using an optic microscope (Zeiss Standard 20, www.zeiss.com) equipped with a camera lucida. Measurements and drawings of head capsules were made with the aid of a stereoscopic microscope (Wild-Heerbrugg M5, www.wild-heerbrugg.com) with a micrometric lens or a camera lucida.

Nomenclature follows Scoble (1992) for eggs; Hinton (1946), Peterson (1962), and Stehr (1987) for larval chaetotaxy and morphology, with modifications proposed by Huertas-Dionisio (2006) for the chaetotaxy of the anal prolegs; and Mosher (1916) and Casagrande (1979) for pupal morphology. Voucher specimens are retained at the Coleção Entomológica Pe. Jesus Santiago Moure, Departamento de Zoologia, Universidade Federal do Paraná, Coleção de Imaturos de Lepidoptera (DZUPIL). Measurements are given ± standard deviation.

## Results

### Biology

**Figures 5–21.**
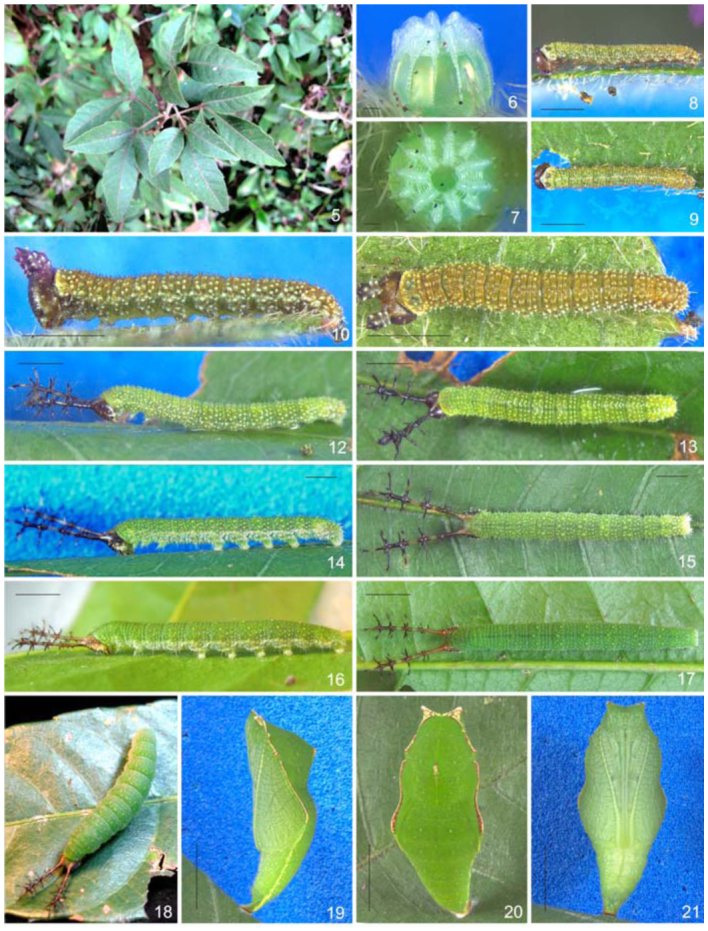
Immature stages and host plant of *Diaethria candrena candrena* (Godart) 5. Host plant, *Allophylus puberulus*. 6–7. Egg. 6. Lateral. 7. Dorsal. 8–9. First instar Larvae. 8. Lateral. 9. Dorsal. 10–11. Second instar larvae. 10. Lateral. 11. Dorsal. 12–13. Third instar larvae. 12. Lateral. 13. Dorsal. 14–15. Fourth instar larvae. 14. Lateral. 15. Dorsal. 16–17. Fifth instar larvae. 16. Lateral. 17. Dorsal. 18. Fifth instar, one day before pupate, dorsal. 19–21. Pupa. 20. Lateral. 21. Dorsal. 22. Ventral. Scale bars: Figures 6–7 = 0.1 mm; Figures 8–11 = 0.5 mm; Figures 12–15 = 1 mm; Figures 16–17 = 0.25 cm; Figures 19– 21 =0.5 cm. High quality figures are available online.

Females of *D*. *c*. *candrena* lay eggs on *Allophylus puberulus* (Figure 5), although eggs are occasionally found on *A*. *edulis*. Both are small trees commonly found in the study site on forest edges and gaps. Eggs are laid on the basal half of the underside of the leaf, often close to the margin. Before hatching, the head of the first instar larvae is dorsally visible through the chorion. First and second instar larvae build frass chains with silk and fecal pellets on the tip of the primary vein of one of the three folioles. When not feeding, first and second instar larvae rest on the frass chains with the head capsule towards the leaf margin. Third to fifth instar larvae rest with the head capsule towards the petiole and with the frons positioned against the leaf surface, thus with the head capsule scoli parallel to the substrate. Later instar larvae move the head capsule scoli sideways, and with up and down movements when disturbed. In the end of the fifth instar the larvae stop eating and swell considerably. The larvae roam around looking for a suitable place to pupate, occasionally abandoning the host plant. The larvae adhere themselves on a silk pad by the anal prolegs, remaining stretched upside down until molt (Figure 18). Pupae are positioned horizontally to the upper side of the leaf and are capable of movement, wobbling vigorously when disturbed.

### Morphology

**Figures 22–24.**
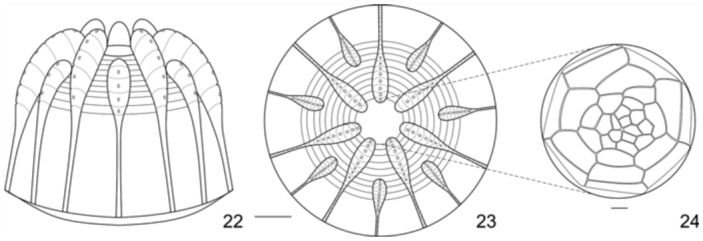
Egg of *Diaethria candrena candrena* (Godart). 22. Lateral. 23. Dorsal. 24. Detail of sculptured area around micropyla, dorsal. Scale bars: Figures 22–24 = 0.1 mm. High quality figures are available online.

Egg. (Figures 6–7): Light green; round, with flattened base and apex; with 14 vertical and 20–22 horizontal ridges. Vertical ridges alternating large and small projections near the apex to form a ‘crown’ (Barbosa et al. 2010) (Figures 22–23); symmetrically arranged around a circular flattened area with rosette—like sculptures (Figure 24). Horizontal ridges noticeable only over the vertical ridges, excluding a series of ten distinct ridges near the apex (Figures 22–23). Micropylae are on the center of the apical sculptured area (Figure 28) and aeropylae on the projections of the vertical ridges (Figure 27). Approximate duration: five days (n = 19). Average diameter 0.61 ± 0.077 mm; height 0.54 ± 0.072 mm (n = 4).

**First instar.** (Figures 8–9): Head capsule dark brown, smooth and slightly bilobated along the epicranial suture; six stemmata roughly placed in a semicircle (Figures 29–30); labrum bilobated (Figure 31); mandibles with round teeth (Figure 32). Body mostly green; yellowish—green on the prothorax and A8– A9+10; with whitish spots scattered on the spiracular area and dark brown setae inserted on roundish dark brown pinnacula. Prothoracic plate dark brown and semicircular (Figure 34); suranal plate indistinct, unevenly sclerotized (Figure 35); thoracic and abdominal proleg plates and ocrea dark brown, abdominal and anal prolegs bearing 10–12 uniordinal and uniserial crochets arranged on a lateral penellipse. Chaetotaxy of the head capsule and body are given by Figures 29–35. Approximate duration: five days (n = 16). Head capsule width: 0.30 ± 0.09 mm (n = 4).

**Second instar.** (Figures 10–11): Head capsule dark brown, with tiny whitish projections and a pair of thick truncated scoli about half the height of the head capsule, one on each side of the epicranial suture (Figure 36). Body dorsal, subdorsal, and supraspiracular areas yellowish—green; subspiracular, subventral, and ventral areas green; with scattered whitish spots on the insertion of the secondary setae; prothoracic plate green; thoracic and abdominal proleg plates and ocrea dark brown. Approximate duration: five days (n = 20). Head capsule width: 0.37 ± 0.097 mm (n = 5)

**Third instar.** (Figures 12–13): Head capsule dark brown, with scattered tiny whitish projections, with slender and branched scoli about three times the height of the head capsule, one on each side of the epicranial suture. Scoli covered by long setae; with tiny anterior spines on the base; two rows of four longer spines on the second and third fourth of the shaft of the scoli, which ends in a ‘crown’ (Barbosa et al. 2010) of five lateral spines and an additional distal spine (Figure 37). Shaft of the scoli yellowish between the second and third rows of spines. Body entirely green but lighter green ventrally, with scattered whitish spots on the insertion of the secondary setae and along the spiracular area; prothoracic plate of the same color as the surrounding areas; thoracic and abdominal proleg plates and ocrea light green. Approximate duration: six days (n = 36). Head capsule width: 0.61 ± 0.076 mm (n = 10)

**Fourth instar.** (Figures 14–15): Head capsule and body generally similar in color and shape to the previous instar, but head capsule with ventral and posterior yellowish—green areas, also along the adfrontal and epicranial suture. Scoli about two and a half times the height of the head capsule; shaft of the scoli also yellowish—green between the anterior tiny spines and the second rows of spines, so as the mid portion of the spines (Figure 38). Body with yellowish round spots on the subdorsal area. Approximate duration: six days (n = 47). Head capsule width: 0.89 ± 0.075 mm (n = 12)

**Fifth instar.** (Figures 16–17): Head capsule and body generally similar in color and shape to the previous instar, but with the posterior half of the head capsule and scoli reddish; with extensive posterior green areas and along the adfrontal and epicranial suture (Figure 39). About one day before pupation, the body swells and becomes uniformly dark green (Figure 18). Approximate duration: 10 days, including about one day in prepupa (n = 48). Head capsule width: 1.41 ± 0.072 mm (n = 8).

**Pupa.** (Figures 19–21; 40–42): Mostly dark green with whitish and yellowish spots dorsally; light green ventrally; brown and yellow markings on the head projections, ridge of mesonotum, basilar tubercle and along the longitudinal ridge; and a thin yellow lateral stripe on A4–A9+10. Pupae rather flattened dorso—posteriorly; head with small projections; prothorax narrower than the rest of the thorax; abdomen conical and A4– A9+10 capable of movement. Head projections lateral and triangular; scape and pedicel dorsal, the former much larger than the latter; antennae flagellum dorsal at first, extending ventrally and posteriorly between the mesothoracic wing cases; eye cases lateral and divided in one rough and other smooth area; frons and clypeus clearly distinguishable from the genae, anterior tentorial fovea slit visible between these two areas; clypeus triangular; mandibles trapezoidal; labium pentagonal, between the mandibles and ventral to the clypeus; galeae extending between the mesothoracic legs beyond the mesothoracic wing cases. Prothorax wide and trapezoidal; mesothoracic spiracle between prothorax and mesothorax; mesothorax dorsally bulged, with a distinct ridge; basalar tubercle triangular and rough; longitudinal ridge ventral to the basalar tubercle, extending posteriorly to the mesothoracic wing cases; mesothoracic wing cases ventral, wing shape and venation visible; prothoracic and mesothoracic legs between the galae and the mesothoracic wing cases, the former approximately two thirds the size of the latter; mesothoracic legs tips visible posterior to the antennae; metatorax ‘M’ shaped; metathoracic wing cases barely discernible between the abdomen and the mesothoracic wing cases. Al–A3 ventrally covered by the thorax; first spiracle not visible; spiracles green and ellipsoidal; spiracles A2 and A3 dorsal, and A4–A8 lateral; A5–A9+10 conical, gradually tapering posteriorly. Genital scar slits almost indiscernible on A9 (males) or A8 and A9 (females); anal scar slit distinct and surrounded by two rough tubercules. Cremaster large and green, directed ventrally and ending on flattened and ventrally bilobated area with several tiny hooks. Approximate duration: 12 days (n = 45). Average height: 1.55 ± 0.12 cm; width: 0.55 ± 0.98 cm (n= 10).

## Discussion

### Host plant use

*Diaethria c*. *candrena* uses exclusively Sapindaceae spp. as host plants. Host plant shift experiments carried out in the laboratory confirm the use of a variety of species of Sapindaceae by *D*. *c*. *candrena*, even ones that immatures of this species were never found on in the field. Under natural or artificial conditions, *D*. *c*. *candrena* never used species of *Celtis* as a host plant. Therefore, the record of Ulmaceae given by Brown (1992) and Beccaloni et al. (2008) need further confirmation. The use of *D*. *c*. *candrena* for *A*. *puberulus* in the present work is probably associated with the host plant use of five other Sapindaceae—feeding Biblidinae recorded on the same site. *Diaethria c*. *candrena* and *D*. *e*. *eluina* (Hewitson) share the same host plants, but the latter is scarcer in the study site (n = 3) (F. Dias personal observation). *Callicore pygas eucale* (Fruhstorfer) also uses *A*. *puberulus* as host plant on the study site; however, this species is more frequently found on *A*. *edulis* and occur in lower densities than *D*. *c*. *candrena* (Dias, unpublished observations). Nevertheless, the segregation of habitat niches within the same host plant species, as seen in other butterflies (e.g., Yamamoto 1981; Queiroz 2002), was not identified on the present study. The other three Sapindaceae—feeding Biblidinae found on the same site, *Temenis laothoe meridionalis*, *Epiphile orea orea,* and *E*. *hubneri* (all Epiphilini according to Wahlberg et al. 2009) all use *Serjania laruotellana* as preferred host plant, and occasionally two further species of *Serjania* (Dias et al. in preparation). As previously noted, the use of Sapindaceae as a host plant is a character shared by the clade Epiphilini+Callicorini (Barbosa et al. 2010).

### Morphologic and taxonomic considerations

Eggs of species of *Callicore* are strikingly similar to those of *Diaethria*, but the former are yellowish and with more noticeable horizontal ridges (Dias et al. in preparation).Additionally, some *Callicore* species such as*C*. pygas eucale lay eggs near the apex of the leaf (Dias et al. in preparation). First and second instar larvae of *Callicore* are nearly indistinguishable from those of *Diaethria*, including prothoracic plate morphology, head capsule and body chaetotaxy. Only from the third instar on can both genera be distinguished, mostly by the head capsule scoli morphology, particularly *C*. *sorana* and *C*. *cynosura* (Teshirogi 2007; AVL. Freitas personal communication). From the first to the third instar known larvae and pupae of *Diaethria* species are virtually indistinguishable from each other. Although very similar, fourth and fifth instar larvae of*D*. clymena janeira and *D*. *clymena meridionalis* are distinguishable from those of *D*. *c*. *candrena* by the presence of a yellow subdorsal stripe along the body, and the presence of developed thoracic scoli (Barbosa et al. 2010). *Diaethria e. eluina* scoli are less developed than those on *D*. *clymena*, but it can also be used to distiguish fourth and fifth instar larvae from the former species from those of *D*. *c*. *candrena* (F. Dias personal observations).

The reduction of body scoli is a condition found on most Callicorini (*Perisama*, *Haematera*, *Callicore*, *Diaethria*) and some other Biblidinae (*Cybdelis*, some *Eunica*) (Freitas et al. 1997; Teshirogi 2007). This reduction is quite extreme on *D*. *c*. *candrena*. Pupae of *Callicore* species are clearly distinguishable from those of *Diaethria*: the former lack head projections and are dorsally colored with a two—tone green pattern. A more comprehensive comparison between morphological and behavioral traits of Epiphilini and Callicorini immatures is given by Barbosa et al. (2010).

Detailed descriptions are of great importance not only to aid in identification of larvae in the field, but they are also fundamental to extract characters for taxonomy and phylogenetic inference (Freitas and Brown 2004; Barbosa et al. 2010). Detailed studies of immature stage morphology and information on host plant use can be helpful to settle certain taxonomic conundrums, and thus should be carefully reported (Dias et al. 2011). It is expected that further information on immature stages of Biblidinae will be of great value (Freitas et al. 1997), especially when information from species of some genera (e.g., *Paulogramma*, *Catacore*, *Mesotenia*, *Antigonis*, *Orophila*) and taxa from speciose genera which immature stages are still unknown becomes available.

**Figures 25–28.**
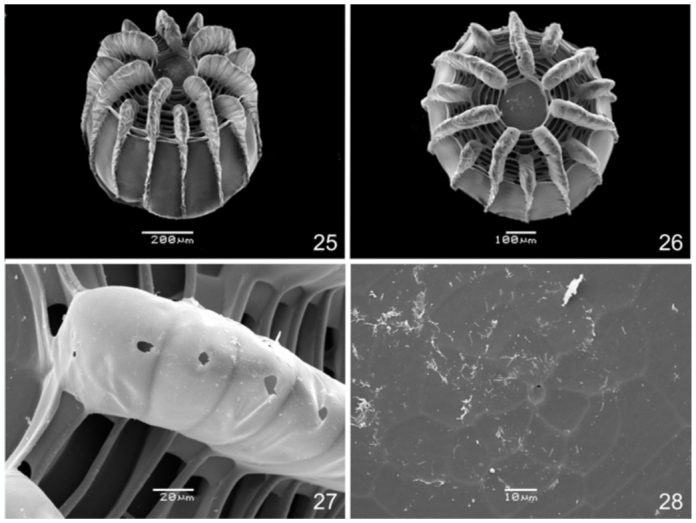
Egg of *Diaethria candrena candrena* (Godart). 25. Dorso-lateral. 26. Dorsal. 27. Aeropylae on vertical ridge, dorsal. 28. Sculptured area around micropylae, dorsal. High quality figures are available online.

**Figures 29–35.**
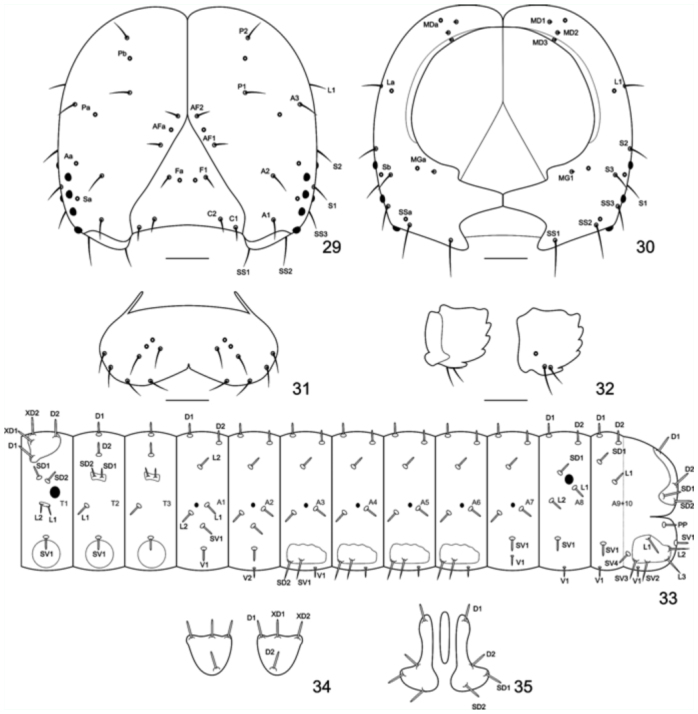
Chaeototaxy of *Diaethria candrena candrena* (Godart). 29–30. First instar head capsule. 29. Anterior. 30. Posterior. 31. Labrum, anterior. 32. Mandibulae, posterior and anterior. 33. Thorax and abdomen schematic representation, lateral. 34. Prothoracic plate, dorsal. 35. Anal plate, dorsal. Scale bars: Figures 29–30 = 0.05 mm; Figures 31–32 = 0.025 mm. High quality figures are available online.

**Figures 36–38.**
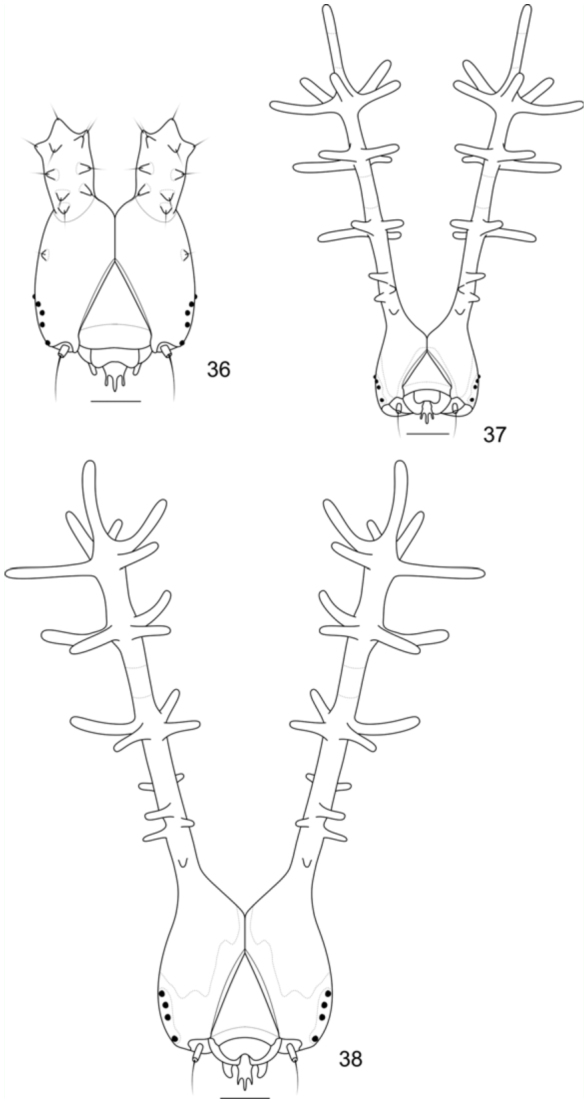
Head capsules of *Diaethria candrena candrena* (Godart), anterior. 36. Second instar. 37. Third instar. 38. Fourth instar. Scale bars: Figure 36 = 0.1 mm; Figures 37–38 = 0.25 mm. High quality figures are available online.

**Figure 39.**
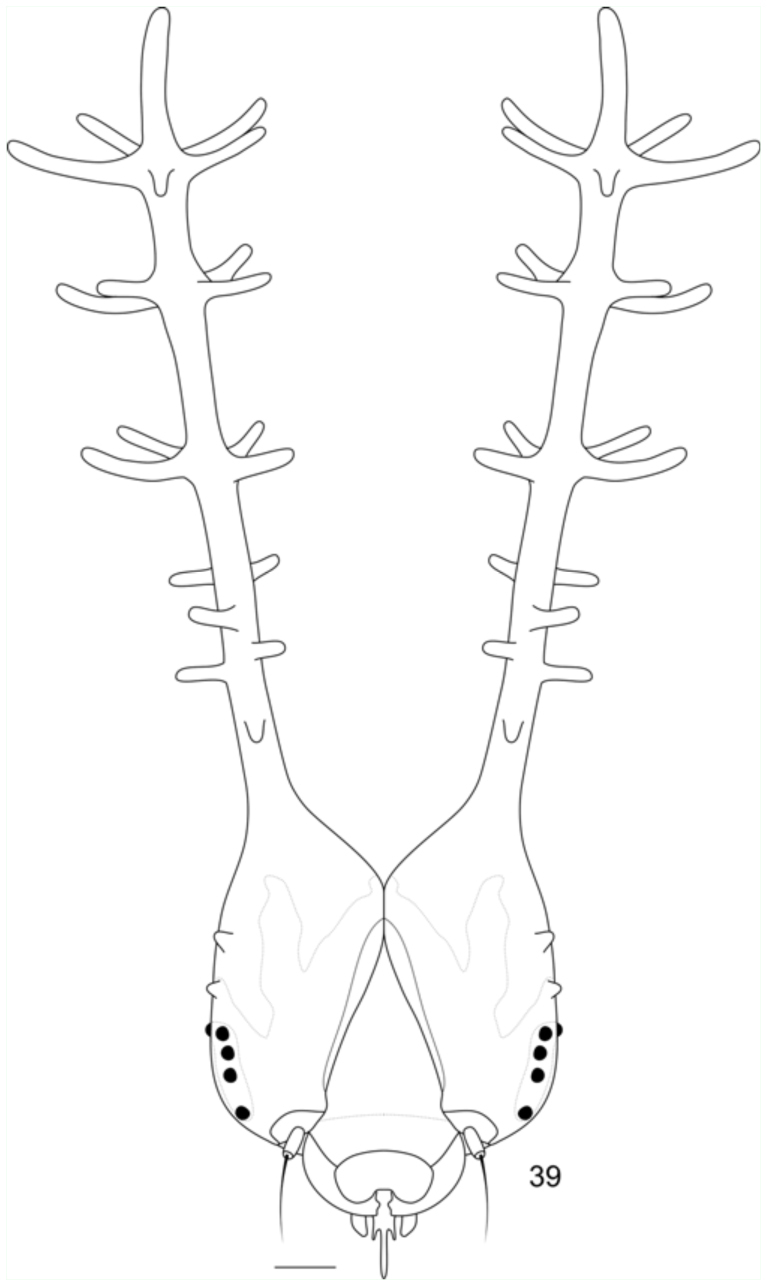
Head capsule of fifth instar *Diaethria candrena candrena* (Godart), anterior. Scale bar: 0.25 mm. High quality figures are available online.

**Figures 40–42.**
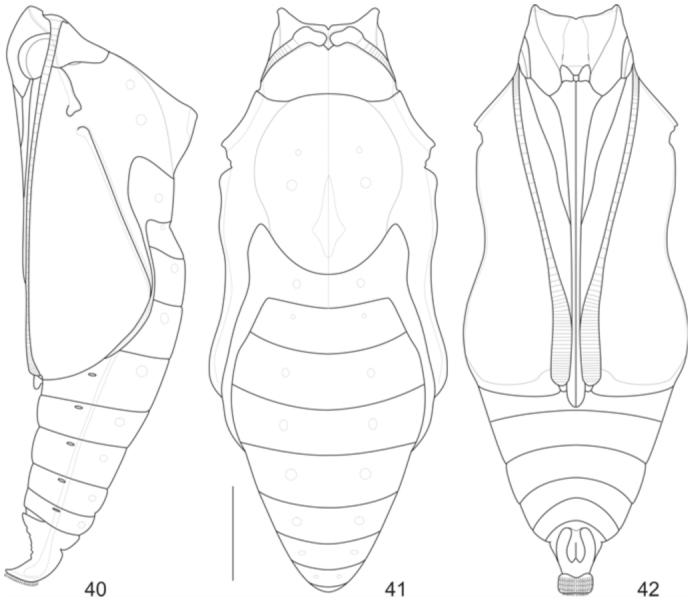
Pupae of *Diaethria candrena candrena* (Godart). 40. Lateral. 41. Dorsal. 42. Ventral. Scale bar: 0.25 cm. High quality figures are available online.
